# Monitoring non-parametric profiles using adaptive EWMA control chart

**DOI:** 10.1038/s41598-022-18381-8

**Published:** 2022-08-22

**Authors:** Saddam Akber Abbasi, Ali Yeganeh, Sandile C. Shongwe

**Affiliations:** 1 Statistics Program, Department of Mathematics, Statistics and Physics, College of Arts and Science, Qatar University, 2713 Doha, Qatar; 2 Statistical Consulting Unit, College of Arts and Science, Qatar University, 2713 Doha, Qatar; 3 Department of Industrial Engineering, Faculty of Engineering, Ferdowsi University of Mashhad, Mashhad, Iran; 4 Department of Mathematical Statistics and Actuarial Science, Faculty of Natural and Agricultural Sciences, University of the Free State, Bloemfontein, 9301 South Africa

**Keywords:** Engineering, Chemical engineering

## Abstract

To monitor the quality of a process in statistical process control (SPC), considering a functional relationship between a dependent variable and one or more independent variables (which is denoted as profile monitoring) is becoming an increasingly common approach. Most of the studies in the SPC literature considered parametric approaches in which the functional relationship has the same form in the in-control (IC) and out-of-control (OC) situations. Non-parametric profiles, which have a different functional relationship in the OC conditions are very common. This paper designs a novel control chart to monitor not only the regression parameters but also the variation of the profiles in Phase II applications using an adaptive approach. Adaptive control charts adjust the final statistic with regard to information of the previous samples. The proposed method considers the relative distance of the chart statistic to the control limits as a tendency index and provides some outcomes about the process condition. The results of Monte Carlo simulations show the superiority of the proposed monitoring scheme in comparison with the common non-parametric control charts.

## Introduction

Employing statistical process control (SPC) scheme for monitoring industrial processes has been widely developed throughout the past few decades. The main aim of this approach is to improve the quality of the products by reducing unfavorable variations. By controlling the manufacturing process of a product, SPC detects internal system inconsistencies and provides solutions to improve the production process with the help of some major tools involving Pareto chart, control chart, cause-and-effect diagram, scatter diagram, check sheet, stratification and histogram^1,2^.

In monitoring quality characteristics of industrial processes, control charts have been considered as the most acceptable and appropriate tool since the 1920s, for both Phase I and II processes. The aim of Phase I is the estimation of process parameters based on an in-control data set and on the other hand, one tries to conduct on-line monitoring of the process parameters in Phase II. So, the major objective in Phase II is to identify where does the process change from IC (in-control) to OC (out-of-control) situation. To evaluate a control chart in Phase II, the well-known criteria, i.e., ARL (average run length) is usually employed. It measures the average number of generated statistics to reach an OC or IC signal (hereafter denoted with *ARL*_*1*_ (*ARL*_*0*_), respectively)^3^. More details about Phase II analysis of SPC could be found in Zou et al.^4^ and Mahmoud et al.^5^.

To develop a control chart in SPC, two major approaches could be utilized. The use of either the univariate or multivariate distribution of the quality characteristics. A user can adopt the state-of-the-art method which investigate the relationship between two or more quality characteristics as a regression model (i.e., the dependent and independent variables could be representative of the process condition). Profile monitoring is usually referred to monitoring of such a regression relationship, that is, investigating the validation of the regression model over time is the main aim^6^. In this subject, different profile shapes (types) such as linear^7–11^, non-linear^12,13^, logistic^14–16^, Poisson^17,18^, circular and cylindrical^19^, mixture profiles^20^ and so forth, have been extended in the SPC field. Since the idea of this paper has been adopted from Phase II monitoring of non-parametric profiles; hence, a short literature review of these types of control charts only has been provided below. More details of profile control charts’ publications prior to the year 2018 can be found in the review paper by Maleki et al.^21^.

### Literature review of non-parametric profiles

Questing the literature revealed that most of the existing profiles control charts are designed in the same way as the quality characteristics’ schemes have been designed. That is, the profiles charts are usually based on some fundamental assumptions (e.g., a parametric distribution) and if the underlying distribution is different, then the conclusion does not hold. As a common assumption in profile monitoring papers, see for instance^7,9,22–24^, parametric schemes constrain the OC form of the profiles to follow the IC formulation. Note, the latter is not always a reasonable assumption because the OC model may not be easily determined (especially in complicated process, such as non-linear models) and it is often invalid in real-life practical applications. Moreover, it may increase the values of *ARL*_*1*_ and non-compatible products (due to a high false alarm rate).

To overcome these problems, several non-parametric control charts have been extended for monitoring quality characteristics. In most of the non-parametric methods, a distribution-free statistic such as Mann–Whitney test is computed for the monitoring purpose^25^. Besides, several other approaches could be found in the related literature^26^. In spite of the fact that there are several non-parametric control charts for monitoring quality characteristics, few studies can be found for non-parametric profiles monitoring, especially in phase II applications. The following paragraphs provide a comprehensive review of the related literature in which the most focuses lie in the researches with different IC and OC models. Hereafter, similar to the main focus of this study, the profiles that have different IC and OC shapes are denoted as non-parametric profiles.

As the fundamental research, Williams et al.^27^ extended five metrics in monitoring non-linear profiles in Phase I based on measuring the deviations from the IC situation. Although these methods created a parametric control chart, they can also be considered as non-parametric if the user does not assign a prespecified distribution of parameters. Considering this work, Zou et al.^28^ developed a non-parametric multivariate EWMA (exponentially weighted moving average) statistic called NEWMA (non-parametric EWMA) for phase II profile monitoring. In this method, the coefficients and standard deviation parameters are firstly scaled and thereafter are taken into account as the previous samples to construct a MEWMA (multivariate EWMA) statistic. On the ground of simulations results of Zou et al.^28^’s paper, it can be counted as the most fundamental control chart in phase II of non-parametric profile monitoring. Some other extensions of this fundamental paper could be found in Qiu and Zou ^29^, Qiu et al.^30^, Hung et al.^31^, Chuang et al.^32^ and Li et al.^33^.

Designing EWMA3 statistics proposed by Kim, Mahmoud and Woodall^7^ considered as one of the most common approach in simple linear profiles (i.e. the EWMA3 statistic by Kim et al.^7^), Zhang et al.^34^ designed the corresponding non-parametric method. By consideration of changing the linear IC model to quadratic form, they compared the EWMA3 approaches with some hypothesis tests including LRT (likelihood ratio test), *F* and *T*^*2*^. The same approach has been utilized in Shang et al.^17^ by considering the binomial and Poisson IC models. Similarly, Zhang et al.^34^ and Zi et al.^35^ developed a non-parametric control chart for monitoring linear profiles, where the Wilcoxon rank estimator was employed (instead of the common least squares approach) and the simulation results showed the favorable properties of this approach.

Because non-parametric methods usually deal with complicated profiles, wavelet transformation has been employed to simplify the complexity of monitoring and enhancing the identification ability of OC sources^36,37^. Also, to ease the computations of wavelets in non-linear profiles, PCA (principal component analysis) techniques has been suggested in Paynabar, Jin and Pacella^38^. Increasing the detection ability of control charts in non-parametric profiles with the aim of machine learning and ensemble learning could be found in Yeganeh et al.^39^ in which more accurate control charts’ techniques are used instead of conventional approaches. Jones et al.^6^ provided some useful guidelines for the implementation of the non-parametric control charts in profile monitoring.

Most previous research on monitoring profiles focused on identical profile structure for OC and IC situations or parametric models; in other words, it is assumed that the OC models are restricted to have the same shape as the IC model. But this assumption may be violated in complicated real-life problems such as image and video processing, large-scale data, robotics, sensors’ surveillance in a way that different shape between IC and OC profiles would be probable^6,17^.

Neglecting this issue leads to two main flaws in the monitoring procedure. First, the estimation of parameters does not fit with the usual methods like ordinary least square (OLS) and secondly, the next challenge occurs when the IC model is nonlinear. In the latter case, some specific estimators are required but they may not have a reasonable performance in case of changing the OC model shape. Zou et al.^28^ discussed these situations and stated that definition of some non-parametric methods is necessary to avoid such similar problems. So, if there is an expectation of different IC and OC shape in a real problem, employing of non-parametric methods seems to be necessary to avoid false signals. It is also obvious that similar to parametric methods in phase II, quick shift detection ability for control charts (which is the main aim of this study) would be valuable.

### Literature review of adaptive control charts in profile monitoring

In the previous literature of profile monitoring, there are several memory-type control charts especially for linear profiles. The idea of ranked set sampling was proposed by Riaz et al.^40^ and Huwang et al.^41^ in which the samples are taken as a memory to be ranked by some criteria. These rank-based monitoring schemes generates very sensitive statistic to small shifts. The Variable Sampling Interval (VSI) idea has also been studied in profile monitoring^14,42–45^. The main idea of VSI is that the samples’ interval is not constant and could be identified based on the OC occurrence probability.

As a general term, these approaches can be referred to as the adaptive control charts in which the sample size, statistic magnitude, interval between the sampling and other parameters are adjusted based on the estimation of the occurred shifts^14,40,42,45–47^. To the best of authors knowledge, except Mohammadzadeh et al.^14^ and Jeong et al.^47^, which have investigated adaptive methods based on the logistic and non-linear profiles, there are no other adaptive methods for complicated models such as the non-linear and non-parametric approaches. The main reason for this may be due to the weak performance and incompatibility with non-parametric limitations of adaptive approaches. Also, the complexity of parameter tunning could stir up some troubles in complicated profile structures.

### The aims and innovations of the study

In the present study, a novel adaptive approach is developed for non-parametric profile monitoring in which there is currently no research based on the adaptive control charts in phase II SPC applications. The proposed approach, whose purpose is to improve the detection ability of NEWMA chart statistics, proposed by Zou et al.^28^, in term of ARL criterion, is developed based on a generally different scheme as compared to older adaptive mentioned methods. It considers the relative distance of NEWMA chart statistics to the OC situations as the major criteria in OC detection and then, converts the obtained relative distance to an EWMA statistics. To reach the best performance in phase II, i.e., minimum ARL_1_ with a constant ARL_0_, a heuristic designing procedure of the parameters is implemented in this paper. The performance evaluation is conducted through extensive non-parametric simulations. In addition to the NEWMA approach as the base statistic, some other non-parametric and adaptive based competitors are used to show its superiority (when integrated with the heuristic design approach). Although the proposed method has been extended based on the NEWMA statistic, it is able to combine with other base statistics in phase II monitoring. The main contributions of this study could be summarized as follows:

- Proposing an adaptive control chart in profile monitoring.
- Reaching the optimum control chart parameters with a heuristic approach.
- Combination of the proposed adaptive approach with NEWMA statistic for monitoring non-parametric profiles.
- Computing the detection ability of the proposed method in term of ARL in comparison with some non-parametric and adaptive conventional methods.

The remainder of this paper is organized as follows: the concept of NEWMA control chart is illustrated in “NEWMA statistic for non-parametric profiles”. The proposed heuristic method of this study is discussed in “The proposed method”. “Simulation results” provides the simulation results for performance evaluation and the corresponding sensitivity analyses is conducted in “Sensitivity analysis”. A real-life example is used to illustrate the implementation of the proposed NEWMA chart in “Illustrative example”. Finally, the concluding remarks and recommendations for the future research are presented in “Conclusion”.

## NEWMA statistic for non-parametric profiles

As we focus on the monitoring of profiles that could be well summarized by non-parametric regression model in phase II, the notations and formulations are extended based on the NEWMA scheme^28^ as the most fundamental control chart in this field. Suppose that for the *j*th (*j* ≥ 1) random sample gathered over time, we have a set of observations entailing a *n*_*j*_*-*variate response vector (***y***_***j***_) and *p* × *n*_*j*_ matrix of explanatory (***X***_***j***_) variables with sample size *n*_*j*_ and for simplicity, it is supposed that the sample sizes are the same in all the profiles, so *n*_*j*_ is replaced with *n*. Also the assumption of the same values in explanatory variable is very common in the related literature (i.e., using ***X*** instead of ***X***_***j***_) so we have ***X* = **(***x***_**1**_,***x***_**2**_,…,***x***_**n**_). By these assumptions, the general IC model can be given by:

1 $$\begin{aligned} & y_{ij} = g_{0} (x_{i} ) + \varepsilon_{ij} , \\ & \varepsilon_{ij} \sim N(0,\sigma_{0}^{2} ), \\ & i = 1,2, \ldots ,n, \\ & j = 1,2, \ldots , \\ \end{aligned}$$

where *g*_*0*_ is the IC regression function and σ_0_^2^ is the IC parameter obtained from phase I or previous information about the process, which are assumed to be known in phase II. Considering ***G***_***0***_ = (*g*_*0*_ (***x***_***1***_), *g*_*0*_ (***x***_***2***_),…, *g*_*0*_ (***x***_***n***_)), to monitor the above model at the *j*th sampling time with the vector of response variables ***Y***_***j***_ = (*y*_*1j*_,*y*_*2j*_,…,*y*_*nj*_), Zou et al.^28^ defined the following statistic:

2 $$\begin{aligned} & {\varvec{Z}}_{{\varvec{j}}} = \frac{{{\varvec{Y}}_{{\varvec{j}}} - {\varvec{G}}_{{\varvec{0}}} }}{{\sigma_{0} }}, \\ & \hat{\sigma }_{j}^{2} = \frac{1}{n}({\varvec{Z}}_{{\varvec{j}}} - {\varvec{WZ}}_{{\varvec{j}}} )^{\prime}({\varvec{Z}}_{{\varvec{j}}} - {\varvec{WZ}}_{{\varvec{j}}} ). \\ \end{aligned}$$

In Eq. (2), ***W*** is a symmetrical *n* × *n* smoothing matrix to be utilized in local linear estimator of response variables and obtained based on the explanatory variables, so it is fixed and constant in each new generated profile. To compute smoothing matrix, a symmetric probability density function and a bandwidth, denoted by *h*_*E*_ are defined and each element of ***W*** are calculated based on them. For brevity, the formulas are not reported here but a MATLAB function for computation of ***W*** is provided by the authors and available upon request. For reaching optimum value of bandwidth, a relationship based on the explanatory variables was introduced in Eq. (10) of Zou et al.^28^. In that Equation, a constant parameter, denoted by *c*, is defined and the simulations are conducted based on different values of *c*.

For developing the MEWMA chart statistic, Zou et al.^28^ first transformed the $\hat{\sigma }_{j}^{2}$ to a standard normal variable:

3 $$\begin{gathered} \tilde{\sigma }_{j} = \varphi^{ - 1} \{ \psi (n\hat{\sigma }_{j}^{2} ;{\varvec{I}}_{n \times n} - {\varvec{V}})\} , \hfill \\ {\varvec{V}} = \user2{W^{\prime}} + {\varvec{W}} - \user2{W^{\prime}W}. \hfill \\ \end{gathered}$$

In this transformation, φ^−1^ is the inverse of the standard normal CDF (cumulative distribution function) and *φ* can be approximated with the chi-square distribution in the way that:

4 $$\begin{aligned} & \frac{{n\hat{\sigma }_{j}^{2} - c_{3} }}{{c_{1} }} \sim \chi_{{c_{2} }}^{2} , \\ & c_{1} = \sqrt {\frac{{tr({\varvec{A}}^{2} )}}{{tr({\varvec{A}}^{3} )}},} \\ & c_{2} = tr({\varvec{A}}^{3} ), \\ & c_{3} = tr({\varvec{A}}) - \sqrt {tr({\varvec{A}}^{2} ).tr({\varvec{A}}^{3} )} , \\ & {\varvec{A}} = {\varvec{I}} - {\varvec{V}}. \\ \end{aligned}$$

Then, ***U***_***j***_, denoted as $({\varvec{Z}}_{{\varvec{j}}} ,\tilde{\sigma }_{j} )^{\prime}$, which is an (*n* + 1)-variate vector and the (*n* + 1)-dimensional symmetric covariance matrix is defined as $${\mathbf{\sum }} = \left( {\begin{array}{*{20}c} {\varvec{V}} & 0 \\ 0 & 1 \\ \end{array} } \right)$$. The proposed EWMA charting statistic then could be calculated as:

5 $$\begin{gathered} {\varvec{E}}_{{\varvec{j}}} = \lambda {\varvec{U}}_{{\varvec{j}}} + (1 - \lambda ){\varvec{E}}_{{{\varvec{j}} - 1}} , \hfill \\ {\varvec{E}}_{{\varvec{0}}} = (0,0, \ldots ,0)_{1 \times (n + 1)} , \hfill \\ 0 < \lambda \le 1. \hfill \\ \end{gathered}$$

And finally, we have a positive chart statistic:

6 $$Q_{j} = \user2{E^{\prime}}_{{\varvec{j}}} {\mathbf{\sum }}{\varvec{E}}_{{\varvec{j}}} ,$$

where *λ* is the EWMA constant (here is equal to 0.2). Considering Eq. (6), an OC signal is triggered if *Q*_*j*_ is located beyond the IC region $\left( {Q_{j} > L\frac{\lambda }{2 - \lambda }} \right)$, where *L* is assigned based on desired *ARL*_*0*_.

## The proposed method

The major idea of the current study is to propose an adaptive approach that is established based on the tendency (relative distance) of obtained statistics to the OC situations. As mentioned in Aly et al.^48^ and Haq and Khoo^49^, an adaptive control chart adjusts the chart statistic based on the shift size of the process in the current time. Following this idea, the proposed tendency or relative distance is computed according to the ratio of the statistic’s distance from upper control limit (UCL) and lower control limit (LCL). So, the tendency of the *j*th sample (*T*_*j*_) is defined as:

7 $${\text{T}}_{j} = \frac{{UCL - Q_{j} }}{{Q_{j} - LCL}}.$$

In NEWMA approach, LCL is set at zero, so the above formula transforms to:

8 $${\text{T}}_{j} = \frac{{UCL - Q_{j} }}{{Q_{j} }}.$$

**Figure 1:**
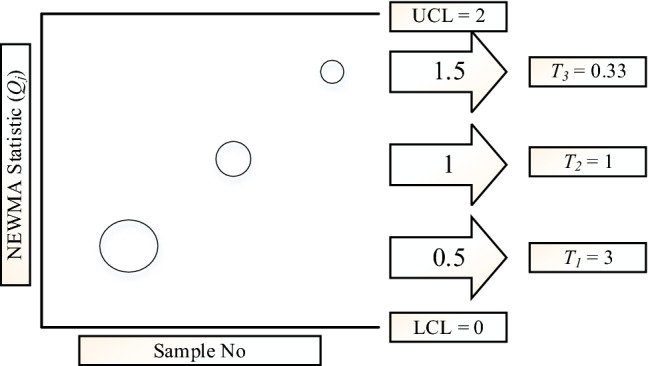
The tendency value for three different NEWMA statistics when LCL and UCL are 0 and 2.

Basically, the lower the charting statistics are, the greater tendency would be. For better understanding, Fig. 1 depicts the tendency values for three different NEWMA statistics when LCL and UCL are 0 and 2, respectively. For example, if the first NEWMA statistic is 0.5, *T*_*1*_ is calculated as 3.

Naturally, such an OC situation in an NEWMA control chart will cause more samples with lower tendency values; in other words, the average of tendencies would be decreased in case an OC situation has been observed. So, the average of tendencies (T^*^_j_) is firstly computed by the following equation and then, it is mapped to a specific rate for applying in an adaptive approach.

9 $${\varvec{T}}^{\user2{*}}_{{\varvec{j}}} \user2{ = }\frac{{{\varvec{T}}_{{\varvec{1}}} + {\varvec{T}}_{{\varvec{2}}} + \cdots + {\varvec{T}}_{{\varvec{j}}} }}{{\varvec{j}}}\user2{.}$$

The aim of this paper is to use the proposed tendency index in Eq. (8) as an auxiliary adaptive approach for adjustment of the final NEWMA statistic. To have a quicker signal detection in OC conditions in the proposed adaptive approach, the NEWMA statistic (*Q*_*j*_) is increased (decreased) when the tendency index (*T*_*j*_) is decreased (increased), respectively. The updating of the NEWMA statistic in the *j*th sample is done by using the tendency rate (defined by *Tr*_*j*_) as follows:

10 $$Q_{j}^{*} = Q_{j} \times Tr_{j} .$$

To have a more sensitive control chart against OC shifts, it is expected that *Tr*_*j*_ to be lower than 1 when T*_j_ has a large value (IC situation); note though, it can be greater than 1 when T*_j_ has a small value (OC situation). Hence, a mapping function or relationship between *Tr*_*j*_ and T*_j_ will be required to use the proposed adaptive approach. This approach has been carried out in previous works by definition of a score function^48,50^, improved estimators^49^, warning limits^42,51^ and so forth.

**Figure 2:**
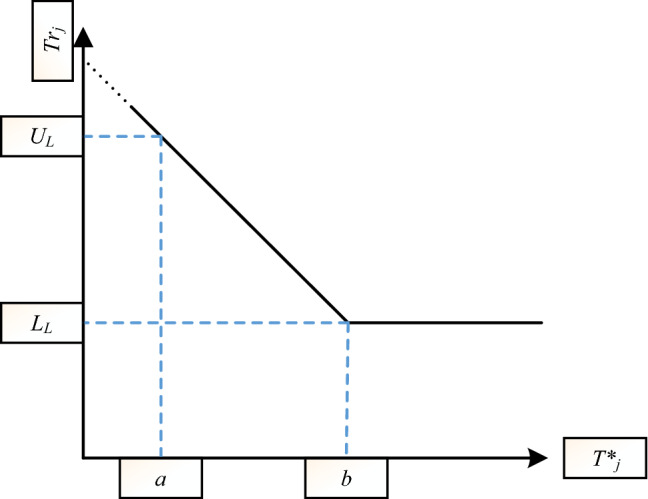
The proposed linear mapping function for obtaining Tr_j_.

To compute *Tr*_*j*_, a completely linear function is not employed in this paper and a hinge function (called mapping function in this paper), as shown in Fig. 2, is utilized. A hinge function hinders very small rates causing very late signalling especially in large shifts so it is not allowed to have smaller tendency rates than lower limit (*L*_*L*_) even the average of tendencies is greater than a predefined value *b*. In addition to *a* and *L*_*L*_, two parameters entailing *B* and upper limit *U*_*L*_ have to be defined for obtaining the mapping function formulation. The dash line means that it is very unlikely to be a sample in that region.

Considering the known values for the *a*, *b*, *L*_*L*_ and *U*_*L*_ (designing of these parameters are discussed later), an OC signal will be triggered when Q*_j_ is greater than UCL. This approach adapts the NEWMA statistic magnitude based on the previous samples in a way that the chart statistic becomes so large to easily reach an OC signal. It is shown through simulations that the proposed approach is able to gain tangibly lower *ARL*_*1*_ values rather than NEWMA chart.

**Figure 3:**
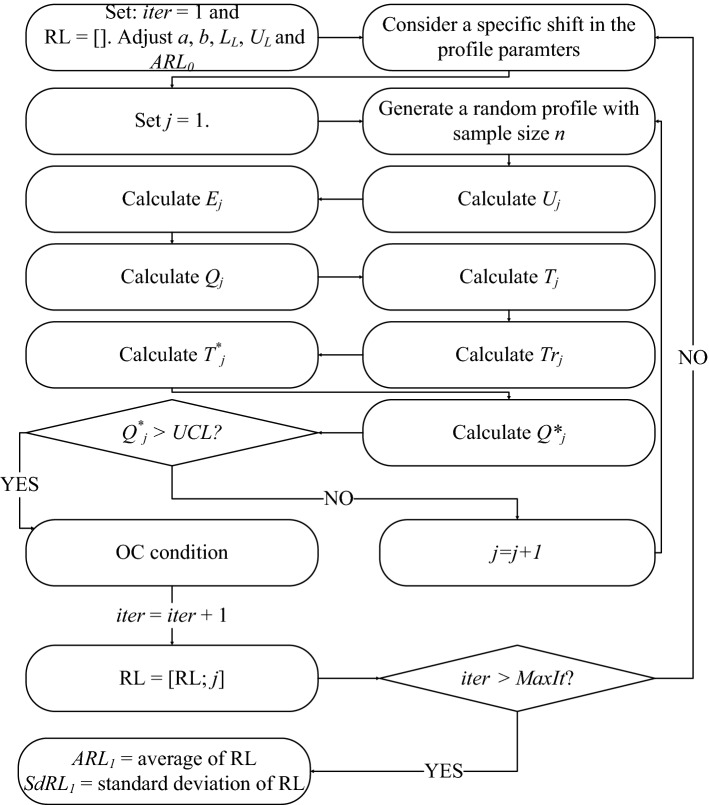
The framework for *ARL*_1_ and *SdRL*_1_ computations in the proposed method.

For better understanding, the procedure of *ARL*_*1*_ simulations is depicted in Fig. 3. In this procedure, the OC profiles are generated to reach a signal in *MaxIt* times and the average of run lengths (signalling time) is reported as *ARL*_*1*_. Similar to *ARL*_*1*_, the standard deviation of run length (*SdRL*_*1*_) is computed for each predefined shift in the process.

In this approach, the designing procedure involves selection a proper value for of *a*, *b*, *L*_*L*_ and *U*_*L*_. Due to complex close form formulation, the control limits are adjusted based on the simulation in this paper; it is a common approach in previous profile monitoring studies^42,45,52^.

The procedure of designing comprises three main directions. First, the value of *UCL* is only related to the *ARL*_*0*_ or equivalently, it is similar with the NEWMA approach. Considering the ration of sample in IC data generation for the coefficients i.e., *a* and *b* is the second one, and keeping the slope of mapping function at a predefined value is the third.

To this aim, 10,000 IC profiles are generated and *T*_*j*_ (*T*_1_, *T*_2_,…,*T*_9999_ and *T*_10000_) are computed. Then, the 45% and 95% quantile of these data are considered as proper values for *a* and *b*. These experimental values have been obtained after several investigations in different IC profiles. To adjust *L*_*L*_ and *U*_*L*_, three following aims should be satisfied:

- *L*_*L*_ < 1 < *U*_*L*_,
− 3 < slope of mapping function < − 1,
- The final *ARL*_*0*_ is supposed to be very close to its desired value.

The rationale behind the first condition is that the proposed method tries to have greater statistics in OC situations. The second condition suggests a reasonable mapping function between *Tr*_*j*_ and T^*^_j_. It has been obtained by several simulation studies in different IC profiles. Finally, the last one is the natural aim of each control chart in phase II application.

By three above conditions, we should be trying to reach the best values of *L*_*L*_ and *U*_*L*_. In the above formula, *L*_*L*_ is expected to be applied in IC condition while the chart statistic will become greater with *U*_*L*_ in OC situation. In other words, the chart statistic is multiplied by a value which is less (greater) than one in large (small) tendencies (or IC (OC) situation), respectively. The second condition has been suggested by several investigations in different IC profiles and the last one is the main criteria for phase II applications. The following steps summarize the designing procedure.

- Step 1: Adjust UCL to reach desired *ARL*_0_. It is the same as the NEWMA approach.
- Step 2: Generate 10,000 IC profiles and store *T*_1_, *T*_2_,…, *T*_9999_ and *T*_10000_.
- Step 3: Calculate the 45% and 95% quantile of these data as *a* and *b*.
- Step 4: Calculate *L*_*L*_ and *U*_*L*_ with regard to the three above conditions.
- Step 5: Obtain the mapping function formula.

## Simulation results

To compare our proposed method, three different simulation setups in which the IC model respectively has the polynomial, exponential and linear form are adjusted here, based on the non-parametric approaches simulated in Zou et al.^28^ and Zhang et al.^34^. The IC model of these scenarios are defined in Table 1 for the *j*th generated profile (*i* = 1, 2,…, *n*).

**Table 1:** The IC model of different scenarios in the jth generated profile.

Scenarios	1	2	3
IC model	$y_{ij} = 1 + 2x_{i} + 3x_{i}^{2} + \varepsilon_{ij}$	$y_{ij} = 1 - e^{{ - x_{i} }} + \varepsilon_{ij}$	$y_{ij} = 3 + 2x^{*}_{i} + \varepsilon_{ij}$ $x^{*}_{i} = x_{i} - \overline{x}$
*x*_*i*_	$\frac{i - 0.5}{n}$	$\frac{i - 0.5}{n}$	$2 + \frac{(8 - 2)(i - 1)}{{n - 1}}$
*n*	20, 40	20, 40	13, 25

Note that in all the models σ_0_ = 1. The competitive methods for the first and second scenarios are NEWMA, NM (naïve MEWMA) and PM (parametric MEWMA). All of these methods have been defined in Zou et al.^28^. It is noteworthy to mention that the NM and PM have not been extended for non-parametric profiles so one would not be surprised with their weak performance. Also, our proposed method has been denoted with ANEWMA hereafter.

Zou et al.^28^ evaluated NEWMA approach under three different setups of bandwidth constant (denoted as *c*) equal to 1, 1.5 and 2. This parameter pertains to *W* in Eq. (2) and for brevity, we only consider the last situation (*c* = 2) in the first and second scenarios. To start the designing procedure (discussed in previous section), the values of UCL are considered as the values reported in Tables 2 and 4 of Zou et al.^28^, i.e. it is computed with $L\frac{\lambda }{2 - \lambda }$. In scenario 3, the competitive methods are KMW (it is the same as EWMA3 approach in Kim et al.^7^) and NEWMA with *c* = 1 (it was not simulated in Zhang et al.^34^ and the results have been obtained based on our simulations). To reach *ARL*_*0*_ equal to 200 in NEWMA approach, *L* is set to 19.58 and 20.75 for *n* = 13 and 25 (the initial values of UCL in our procedure). It is worthwhile to mention that Zhang et al.^34^ did not propose a clear manner for determination of control limits of KMW. But we speculate that they adjusted each separate chart to reach the same *ARL*_*0*_. Note that simulations were not conducted for the case of *n* = 5, this is due to the inability of NEWMA method to signal in some shifts when dealing with small sample sizes (for more details, see Remark 1 in Zou et al.^28^).

We tried to define similar models as those in Zou et al.^28^ and Zhang et al.^34^; however, due to some inconsistency in the results of second scenario in Zou et al.^28^, there are some differences in this case. The OC models in scenario 1 are defined as:

I. $$y_{ij} = b_{0} + b_{{1}} x_{i} + b_{{2}} x_{i}^{{2}} + e_{ij}$$.
II. $$y_{ij} = b_{0} + b_{{1}} x_{i} + b_{{2}} x_{i}^{{2}} + b_{{3}} x_{i}^{{3}} + e_{ij}$$.
III. $$y_{ij} = {1} + {2}x_{i} + {3}x_{i}^{{2}} + b_{{1}} {\text{sin}}({\text{2p}}b_{{2}} x_{i} ) \, + e_{ij}$$.

In this scenario, six different OC shifts have been employed based on Table 2.

**Table 2:** The OC shifts in scenario 1.

Shift type	Model (I)				Model (II)				Model (III)		
	*β*_0_	*β*_1_	*β*_2_	*σ*	*β*_0_	*β*_1_	*β*_2_	*σ*	*β*_1_	*β*_2_	*σ*
(i)	1.0	2.0	3.1	1.0	0.8	4.4	− 3.0	1.0	0.1	1.0	1.0
(ii)	1.0	2.1	3.1	1.0	0.8	4.4	− 3.0	1.0	0.2	1.0	1.0
(iii)	1.1	2.1	3.1	1.0	0.8	4.4	− 3.2	1.0	0.2	0.8	1.0
(iv)	1.0	2.0	3.0	1.0	1.0	4.4	− 3.2	1.0	0.2	1.3	1.0
(v)	1.0	2.0	3.0	0.7	0.8	4.4	− 3.0	1.1	0.3	1.5	1.0
(vi)	1.1	2.1	3.1	1.1	0.8	4.5	− 3.0	1.1	0.3	1.5	1.1

In the second scenario, the OC models are:

I. $$y_{ij} = { 1 } - b_{{1}} {\text{exp}}( - x_{i}^{{b}{2}} ) \, + e_{ij}$$.
II. $$y_{ij} = { 1 } - {\text{ exp}}\left( { - x_{i} } \right) \, + b_{{1}} {\text{cos}}({\text{p}}b_{{2}} \left( {x_{i} {-} \, 0.{5}} \right)) \, + e_{ij}$$.
III. $$y_{ij} = {1} + b_{{1}} {-}{\text{ exp}}\left( {x_{i} } \right) + b_{{2}} x_{i}^{{2}} + e_{ij}$$.

In this scenario, six different OC shifts have been employed based on Table 3.

**Table 3:** The OC shifts in scenario 2.

Shift type	Model (I)			Model (II)			Model (III)		
	*β*_1_	*β*_2_	*σ*	*β*_1_	*β*_2_	*σ*	*β*_1_	*β*_2_	*σ*
(i)	1.00	1.30	1.00	0.20	3.00	1.00	0.05	0.01	1.00
(ii)	1.00	1.50	1.00	0.30	3.00	1.00	0.05	0.05	1.00
(iii)	1.10	1.00	1.00	0.20	2.00	1.00	0.05	0.00	1.05
(iv)	1.30	1.00	1.00	0.30	2.00	1.00	0.00	0.30	1.00
(v)	1.20	1.00	1.10	0.20	4.00	1.10	0.00	0.10	1.10
(vi)	1.00	1.20	0.70	0.20	4.00	1.30	0.50	0.00	1.10

In the third scenario, Zhang et al.^34^ supposed that the IC model changed to quadratic form by adding the $\gamma (x_{i}^{*2} - \eta )$ term to the IC model in the way that *γ* = *δσ*. Note that the EWMA constant is assumed equal to 0.2 in all scenarios.

Considering *ARL*_*0*_ equal to 200, the values of UCL in each scenario based on the Zou et al.^28^’s method (NEMWA approach) are reported in Table 4. Note that the results of first and second scenario are the same as Tables 2 and 4 in Zou et al.^28^. Also, the design parameters for each scenario were gathered in Table 4.

**Table 4:** The control chart parameters in each scenario (*ARL*_*0*_ = 200).

Scenario	1		2		3	
*n*	20	40	20	40	13	25
*c*	2	2	2	2	1	1
UCL	1.74	1.83	1.74	1.83	2.18	2.31
*a*	2	1.99	2	1.99	1.71	1.55
*b*	4	3.98	4	3.98	3.18	2.87
*L*_*L*_	0.65	0.54	0.65	0.54	0.45	0.47
*U*_*L*_	4.95	3.7	4.95	3.7	2	1.84
Intercept	9.25	6.86	9.25	6.86	3.78	3.44
Slope	− 2.15	− 1.59	− 2.15	− 1.59	− 1.04	− 1.04

In the following subsections, our proposed method is compared with other competitors based on the *ARL*_*1*_ criteria in each of the predefined scenarios.

### The simulated ARL_1_ values for scenario 1

The results of *ARL*_*1*_ with respect to the above OC models are gathered in Table 5. These results reveal that the proposed method (ANEWMA) is effective for all of the shifts. For large shifts in the process parameter, its performance is roughly better than competing methods. As can be seen in the results of model (II) and (III), the more complicated the OC model is, the better the outperformance.

**Table 5:** *ARL*_*1*_ values for different shifts in scenario 1.

Model		*n* = 20						*n* = 40					
		NEWMA			NM	PM	ANEWMA	NEWMA			NM	PM	ANEWMA
		*c* = 1	*c* = 1.5	*c* = 2				*c* = 1	*c* = 1.5	*c* = 2			
I	(i)	150.10	144.70	140.00	171.30	137.10	**81.54**	120.60	115.30	110.10	162.30	103.10	**55.43**
	(ii)	66.60	60.80	56.70	98.20	54.20	**29.53**	37.90	34.00	31.80	79.40	29.20	**16.43**
	(iii)	20.70	18.30	17.50	33.10	16.50	**9.54**	11.20	10.30	9.80	22.40	9.10	**6.56**
	(iv)	30.50	27.40	27.00	43.70	26.50	**11.64**	17.50	16.20	15.50	30.50	14.80	**7.93**
	(v)	8.20	6.60	5.90	*	5.30	**4.64**	4.20	3.70	3.50	*	3.20	**2.69**
	(vi)	12.5	11.4	11	17.3	10.8	**6.92**	7.4	7.00	6.7	11.8	6.3	**4.75**
II	(i)	104.60	104.20	113.40	132.20	199.70	**48.64**	66.50	64.00	67.50	114.20	200.10	**25.63**
	(ii)	89.40	87.50	92.50	119.20	154.10	**38.75**	55.20	50.30	52.10	99.30	120.20	**24.32**
	(iii)	78.80	75.70	79.20	109.30	121.00	**29.15**	45.00	42.40	42.80	89.30	84.90	**16.36**
	(iv)	24.20	22.10	21.10	38.90	22.80	**11.54**	12.70	11.60	11.20	26.20	12.20	**7.53**
	(v)	24.50	23.70	22.60	35.80	25.70	**10.52**	13.90	13.00	12.80	24.40	14.00	**7.28**
	(vi)	22.4	20.60	20.30	32.30	22.50	**8.51**	12.60	12.00	11.60	21.70	12.60	**5.52**
III	(i)	106.80	103.10	102.30	137.20	119.60	**48.53**	69.90	66.70	66.20	122.00	81.30	**35.27**
	(ii)	37.80	35.60	34.80	60.10	47.10	**15.32**	20.00	18.40	18.00	43.10	43.10	**9.03**
	(iii)	35.80	32.20	31.30	57.70	31.50	**13.64**	19.00	16.90	16.50	40.60	40.60	**8.98**
	(iv)	37.30	36.20	38.20	58.10	51.80	**15.01**	19.50	18.30	18.60	41.70	41.70	**10.41**
	(v)	18.00	18.30	20.40	29.50	27.40	**9.93**	9.70	9.50	10.00	18.30	18.30	**6.43**
	(vi)	11.2	11.1	11.4	15.5	13.4	**5.89**	6.8	6.6	6.6	10.6	10.6	**4.9**

Best values are in bold.

*Denotes that the related approach has not been able to detect that type of shift.

### The simulated ARL_1_ values for scenario 2

Three different types of OC models with six different shifts are defined in this subsection based on Table 3 (it is the same as Table 3 in Zou et al.^28^). We may be asked about the changing of the third OC model from Zou et al.^28^’s proposed model $\left( {y_{ij} = \frac{1}{{1 + \beta_{1} x_{i}^{{\beta_{2} }} }} + \varepsilon_{ij} } \right).$ This is due to our inability to produce the same results with Zou et al.^28^’s paper. We even re-simulated the results of the NM method (PM was not able to detect these types of shifts) to make sure there was nothing wrong with our simulations, but unfortunately those results were also not obtained. It may be possible that the third model was written incorrectly in that paper.

The results of *ARL*_*1*_ with respect to the above OC models are gathered in Table 6. These results reveal that the proposed method is effective for all of the shifts and the same conclusions with scenario 1 can be drawn and only in few cases, ANEWMA is not the best approach.

**Table 6:** *ARL*_*1*_ values for different shifts in scenario 2.

Model		*n* = 20					*n* = 40				
		NEWMA			NM	ANEWMA	NEWMA			NM	ANEWMA
		*c* = 1	*c* = 1.5	*c* = 2			*c* = 1	*c* = 1.5	*c* = 2		
I	(i)	144.50	139.90	134.20	168.30	**100.42**	110.30	103.10	100.60	155.00	**54.31**
	(ii)	99.10	93.00	86.20	131.20	**25.64**	63.80	58.70	55.10	113.30	**24.85**
	(iii)	113.70	109.10	102.20	143.00	**52.61**	79.50	72.70	67.40	129.10	**35.79**
	(iv)	19.90	17.80	16.60	31.80	**9.92**	10.60	9.80	9.40	21.00	**6.93**
	(v)	17.90	16.60	15.70	25.40	**7.68**	10.30	9.70	9.20	17.10	**6.92**
	(vi)	8.2	6.5	5.8	*	**4.46**	4.2	3.70	3.5	*	**3.03**
II	(i)	39.40	40.90	45.90	59.30	**21.41**	20.30	20.10	21.40	43.30	**12.41**
	(ii)	17.90	18.10	20.60	27.10	**9.83**	9.70	9.40	9.90	18.40	**7.52**
	(iii)	37.90	35.20	34.40	60.20	**18.62**	20.00	18.40	17.80	43.40	**10.19**
	(iv)	17.30	16.00	15.50	27.00	**9.01**	9.40	8.80	8.70	18.30	**6.38**
	(v)	17.40	17.80	19.00	24.10	**8.42**	9.80	9.90	10.30	16.10	**7.93**
	(vi)	5.4	5.00	4.90	8.50	**2.85**	3.50	3.30	3.30	6.00	**1.91**
III	(i)	136.43	128.94	120.98	156.64	**89.42**	108.31	96.15	92.94	146.81	**50.33**
	(ii)	102.42	105.19	108.32	148.42	**49.67**	75.42	68.42	66.31	125.40	**27.53**
	(iii)	64.09	61.78	63.81	71.51	**23.62**	39.42	39.60	36.51	55.22	**19.32**
	(iv)	42.41	38.11	35.63	66.27	**16.41**	21.42	19.91	19.51	50.19	**11.51**
	(v)	27.49	26.42	25.19	41.62	**11.51**	15.89	14.79	13.91	29.42	**9.64**
	(vi)	4.91	3.81	**3.79**	5.5	3.85	2.94	2.74	**2.65**	4.20	2.67

Best values are in bold.

### The simulated ARL_1_ values for scenario 3

The results of *ARL*_*1*_ for these types of shifts for *n* = 13 and 25 are reported in Tables 7 and 8, respectively. In these tables, the results of EWMA3 approach are shown with KMW notation.

**Table 7:** *ARL*_*1*_ values for different shifts in scenario 3 when *n* = 13.

*η*	*δ*								Method
	0.025	0.050	0.075	0.10	0.15	0.20	0.30	0.50	
0.50	87.94	25.09	11.79	7.24	4.18	2.99	2.07	1.20	NEWMA
	99.88	33.87	15.33	9.21	4.93	3.38	2.19	1.31	KMW
	**42.22**	**17.53**	**8.66**	**6.67**	**3.91**	**2.31**	**1.37**	**1.00**	ANEWMA
1.00	95.02	28.08	12.93	8.14	4.63	3.28	2.20	1.40	NEWMA
	115.12	46.76	21.62	12.58	6.30	4.16	2.52	1.61	KMW
	**62.73**	**17.50**	**10.47**	**6.87**	**4.74**	**2.69**	**1.54**	**1.00**	ANEWMA
1.50	104.03	36.18	15.57	9.32	5.08	3.66	2.36	1.60	NEWMA
	135.00	64.18	31.71	18.38	8.47	5.24	2.94	1.80	KMW
	**59.53**	**19.70**	**12.06**	**8.59**	**4.79**	**2.94**	**1.71**	**1.10**	ANEWMA
2.00	107.70	36.63	17.63	10.51	5.44	3.85	2.52	1.75	NEWMA
	157.66	91.66	50.91	29.04	12.40	6.91	3.39	1.88	KMW
	**72.61**	**23.69**	**11.78**	**8.82**	**5.11**	**3.38**	**1.85**	**1.24**	ANEWMA
2.50	119.04	42.27	20.19	11.17	6.08	4.10	2.66	1.86	NEWMA
	173.51	124.74	78.02	46.93	18.48	9.00	3.77	1.91	KMW
	**77.61**	**24.98**	**13.49**	**9.24**	**5.88**	**3.80**	**1.93**	**1.43**	ANEWMA
3.00	120.81	47.94	21.16	12.68	6.33	4.30	2.78	1.89	NEWMA
	185.24	150.37	108.27	69.96	25.26	10.58	3.87	**1.42**	KMW
	**82.22**	**25.85**	**13.78**	**9.84**	**6.34**	**4.19**	**2.26**	1.45	ANEWMA
3.5	116.57	49.59	22.12	12.53	6.62	4.43	2.81	1.91	NEWMA
	187.90	163.05	123.14	78.84	27.55	10.89	3.93	1.91	KMW
	**85.98**	**29.03**	**14.66**	**10.69**	**6.16**	**4.05**	**2.18**	**1.46**	ANEWMA
4	115.99	47.63	21.02	11.82	6.30	4.36	2.74	1.89	NEWMA
	186.84	153.71	108.74	70.44	24.92	10.56	3.91	1.91	KMW
	**74.27**	**28.96**	**14.49**	**9.39**	**6.45**	**4.03**	**2.21**	**1.46**	ANEWMA
4.5	118.92	46.69	19.82	11.84	6.16	4.19	2.64	1.84	NEWMA
	172.67	122.59	78.28	46.87	18.70	8.91	3.74	1.91	KMW
	**75.61**	**26.36**	**12.49**	**9.29**	**6.23**	**3.35**	**2.03**	**1.32**	ANEWMA
5	113.03	40.75	17.88	10.25	5.53	3.90	2.52	1.77	NEWMA
	155.49	91.49	50.08	28.94	12.27	6.92	3.42	1.88	KMW
	**65.43**	**21.97**	**12.68**	**8.92**	**5.50**	**3.44**	**1.79**	**1.31**	ANEWMA

Best values are in bold.

**Table 8:** *ARL*_*1*_ values for different shifts in scenario 3 when *n *= 25.

*η*	*δ*								Method
	0.025	0.05	0.08	0.10	0.15	0.20	0.30	0.35	
0.50	59.11	16.09	8.09	5.27	3.22	2.37	1.86	1.00	NEWMA
	73.75	21.14	10.06	6.29	3.64	**2.60**	**1.83**	**1.02**	KMW
	36.37	13.24	8.04	5.18	2.76	2.62	2.04	1.24	ANEWMA
1.00	71.02	19.16	8.99	5.77	3.53	2.61	1.94	1.02	NEWMA
	94.56	31.58	14.49	8.47	**4.64**	**3.20**	**2.10**	**1.16**	KMW
	**38.34**	**14.64**	**8.91**	**5.93**	4.81	3.52	2.63	1.52	ANEWMA
1.50	78.50	22.10	10.08	6.63	3.88	**2.83**	**2.01**	1.09	NEWMA
	119.29	48.61	22.36	13.08	6.44	4.15	2.46	**1.41**	KMW
	**48.57**	**15.63**	**9.14**	**7.13**	**3.24**	3.77	2.31	1.46	ANEWMA
2.00	88.80	24.91	11.58	7.15	4.22	3.05	2.08	**1.23**	NEWMA
	145.96	78.33	39.65	22.50	9.63	5.60	2.86	1.56	KMW
	**57.30**	**16.85**	**9.88**	**7.11**	**3.41**	**2.06**	**1.77**	1.28	ANEWMA
2.50	92.78	28.77	12.98	7.78	4.54	3.27	2.16	1.37	NEWMA
	169.95	117.23	72.62	41.92	15.32	7.25	3.11	1.57	KMW
	**58.64**	**18.10**	**10.48**	**7.85**	**3.86**	**2.28**	**1.15**	**1.11**	ANEWMA
3.00	101.36	27.81	13.66	8.21	4.68	3.34	2.20	1.46	NEWMA
	185.12	153.22	105.28	64.41	20.00	8.18	3.18	1.58	KMW
	**69.71**	**19.37**	**11.18**	**8.94**	**4.52**	**2.49**	**1.29**	**1.23**	ANEWMA
3.5	103.04	30.46	13.48	8.28	4.69	3.32	2.21	1.44	NEWMA
	186.83	150.01	107.13	64.76	20.13	8.10	3.16	1.57	KMW
	**63.45**	**19.58**	**10.57**	**8.38**	**4.29**	**2.72**	**1.37**	**1.21**	ANEWMA
4	89.06	28.81	12.70	7.85	**4.53**	**3.21**	**2.16**	**1.38**	NEWMA
	170.40	118.56	72.39	42.00	15.60	7.29	3.10	1.58	KMW
	**56.52**	**18.82**	**10.44**	**8.49**	4.54	3.35	2.19	1.44	ANEWMA
4.5	85.83	26.00	11.50	7.15	4.19	3.01	**2.08**	**1.22**	NEWMA
	147.63	77.19	39.26	22.03	9.67	5.57	2.87	1.56	KMW
	**60.10**	**16.76**	**9.62**	**7.22**	**4.12**	**2.84**	2.25	1.24	ANEWMA
5	80.03	22.03	10.38	6.44	**3.84**	**2.84**	**2.02**	**1.10**	NEWMA
	117.41	48.97	22.55	12.96	6.38	4.14	2.45	1.43	KMW
	**47.53**	**15.93**	**8.72**	**6.63**	3.87	2.95	2.15	1.21	ANEWMA

Best values are in bold.

The superiority of the NEWMA to KMW is predictable due to the linear structure of the KMW control chart. Also, NEWMA has better performance than the other competitors reported in Zhang et al.^34^ when *n* = 13. However, when *n* = 25, the ANEWMA outperformed the other competitors in small shifts only, while the NEWMA is the best for the detection of moderate and large shifts. Since the ANEWMA naturally improves the detection ability of NEWMA, it gives logically the best performance for the detection of all the shifts.

### The simulated SdRL_1_ values for scenario 3

**Figure 4:**
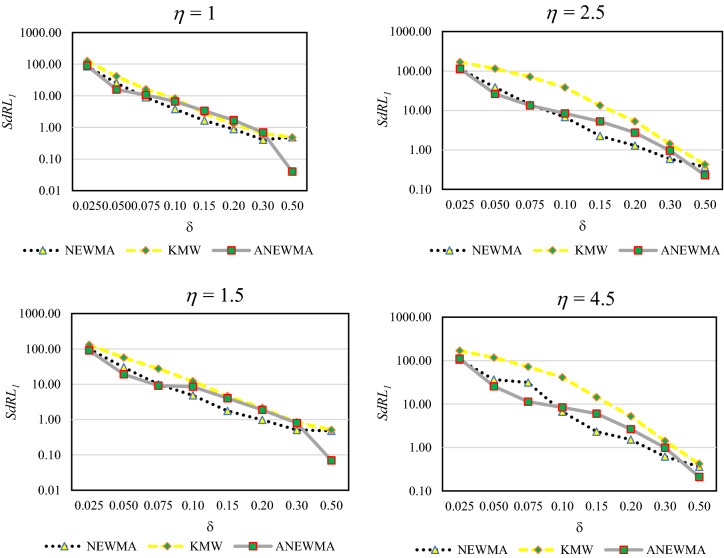
The results of *SdRL*_*1*_ for the scenario 3.

To evaluate control charts in phase II, *SdRL*_*1*_ could be used in addition to *ARL*_1_^53–55^ and such a proper approach is expected to have lower *SdRL*_*1*_ values. Figure 4 illustrates the *SdRL*_1_ values for ANEWMA, NEWMA and KMW schemes. We can see that the proposed approach was able to reduce *SdRL*_1_ for ANEWMA chart in comparison with NEWMA chart. The same results have been generated by other scenarios but they have been omitted for the sake of brevity.

## Sensitivity analysis

In this section, two different simulations are provided to show the superiority of the proposed method. In the first simulation, two conventional adaptive approaches are prepared for monitoring non-parametric profiles. In the first approach, following the Zou et al.^23^ and Mohammadzadeh et al.^14^, VSI scheme is implemented and the proposed adaptive method in Haq^46^ is the second one. These methods hereafter called VSINEWMA and HQNEWMA are incorporated with NEWMA statistic to have a fair comparision. For brevity, the details of designing have not been provided here.

To show the effectiveness of the proposed adaptive method, the NM statistic^28^ are combined with the proposed adaptive approach [denoted with ANM (Adaptive NM)] in the second simulation, the purpose of which is to compare the detection ability of ANM and NM. To this aim, tendency rate based on Eqs. (9) and (10) is multiplied by NM statistic instead of NEWMA statistic. The designing procedure is the same as the ANEWMA and for the brevity the details are neglected. Also, the results of the first scenario are only reported due to page limit.

### Comparison of ANEWMA with other adaptive approaches

**Figure 5:**
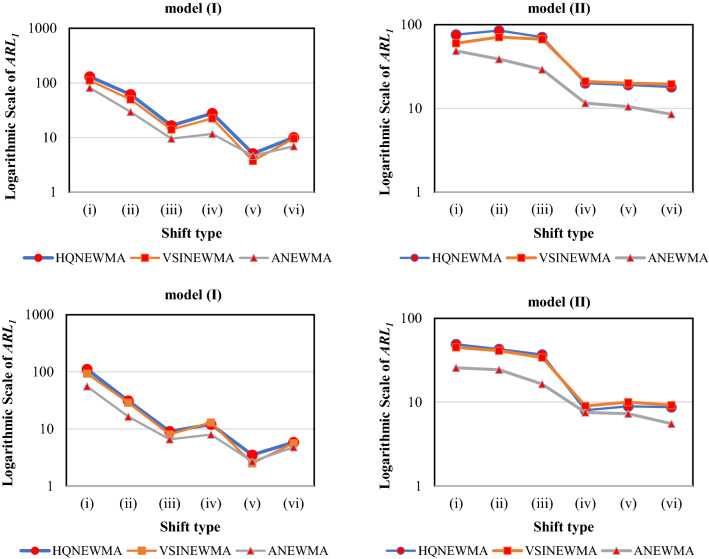
The results of *ARL*_*1*_ for ANEWMA, VSINEWMA and HQNEWMA methods (the figures on top and bottom are for *n* = 20 and 40 respectively).

Figure 5 reports the ARL_1_ values of the first scenario for model (I) and (II) when *n* is 20 and 40. Note that the ANEWMA results have been gathered from Table 5. The superiority of the proposed method over two adaptive competitors is obvious from the following simulations. Except the shift type (v) in model (I) when *n* = 20, ANEWMA has the minimum ARL_1_ value in all of the simulations. Also, VSINEWMA is nearly better than the HQNEWMA in these simulations.

### The effect of proposed adaptive approach in combination with NM statistic

**Figure 6:**
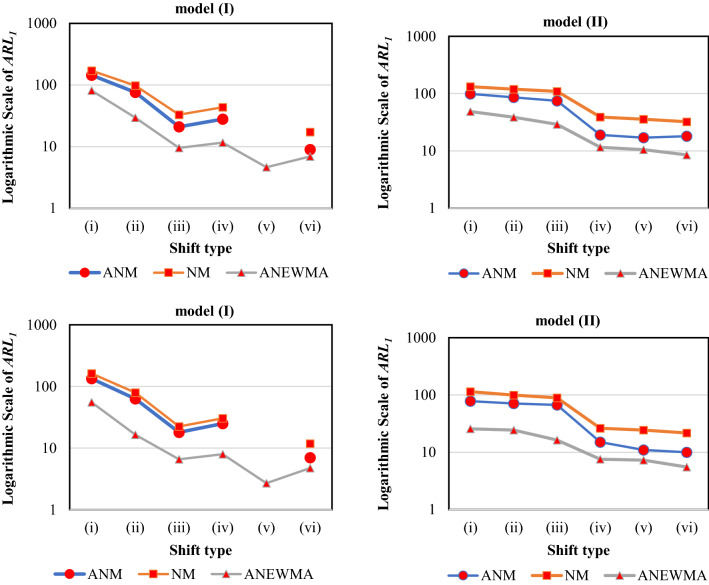
The results of *ARL*_*1*_ for ANEWMA, NM and ANM methods (the figures on top and bottom are for *n* = 20 and 40 respectively).

Figure 6 reports the ARL_1_ values of the first scenario for model (I) and (II) when *n* is 20 and 40. In all of the shifts, ANM had the better performance over NM control chart which is an indicator of the capability of the proposed adaptive approach. Also, ANEWMA outperformed ANM for all the shifts which is due to the better detection ability of NEWMA, compared to NM. The supririty of the proposed method over two adaptive competitors is obvious from the following simulations. Except the shift type (v) in model (I) when *n* = 20, ANEWMA has the minimum ARL_1_ value in all of the simulations. Also, VSINEWMA is better than HQNEWMA in these simulations.

The above two simulations revealed two main findings. First, the proposed method not only outperformed non-parametric and parametric control charts but also had the superiority over conventional adaptive methods. As the second finding, it may be possible to reach better performance than the Haq^46^’s method by using the optimisation of the parameters regarding the non-parametric profiles; however, it is not in the scope of this paper and would be valuable for the future research.

## Illustrative example

A real case is provided in this section to show the practical application of the proposed scheme. It was selected from the semiconductor device fabrication in which there is a deep reactive ion-etching process. The aim of this process is to check an etched wafer by an electron microscopy scanner in scientific labs. Employing of this wafer is needed to run complicated mechanical–chemical reactions for a complex automotive system^23,24,28,56^. In these reactions, one key factor is to determine the profile of a trench in the system because it can change the outcome of the downstream operations and also the final quality of the product.

To establish a suitable monitoring approach for such a trench profile, Zou et al.^23^ suggested a new method based on the profile monitoring control charts. By using the Scanning Electronic Microscope (SEM) data rather direct measurements, they were able to reach at a proper monitoring scheme.

They suggested an IC polynomial model (Eq. 11) for this problem with the following transformation *z*_*i*_ = *x*_*i*_ and $z_{i}^{2} = x_{i}^{2} - \sum\nolimits_{i = 1}^{11} {\frac{{x_{i}^{2} }}{11}}$.

11 $$\begin{aligned} & y_{ij} = 1.55 + 0z_{i} + 0.62z_{i}^{2} + \varepsilon_{ij} , \\ & i = 1,2, \ldots ,11;\quad j = 1,2, \ldots , \\ & x_{i} = - 2.5:0.5:2.5, \\ & \varepsilon_{ij} \sim N(0,0.16), \\ \end{aligned}$$

where the dependent and independent variables are the shape of the profile. Due to complexity of getting real data, the new samples were generated by simulations in Zou et al.^23^. To have an *ARL*_*0*_ equal to 370, *UCL*_*NEWMA*_ was set at 2.01 (*L* = 18.00) when *c* = 1.5^28^. So, the design parameters (as described in “The proposed method”) are *a* = 2.3, *b* = 4.6, *L*_*L*_ = 0.46, and *U*_*L*_ = 2.85. The mapping function’s intercept and slope were obtained as 5.24 and − 1.04 and 1.41 by the proposed designing scheme.

By changing the standard deviation to 0.48 from 0.4 (it is equal to a shift magnitude of 1.2σ), the OC profiles were generated. Table 9 gathers the details of the 15 OC generated profiles. As can be seen, NEWMA was able to trigger an OC signal in the 15th sample while ANEMWA only needed 7 samples to detect this shift.

**Table 9:** The details of 15 OC generated profiles in illustrative example.

*j*	*y*_*ij*_											*Q*_*j*_	*T*_*j*_	*Tr*_*j*_	Q*_j_
1	3.88	2.88	1.50	− 0.30	− 0.10	− 0.14	1.03	1.06	1.37	3.11	3.37	0.33	5.11	0.46	0.15
2	3.71	3.16	2.12	0.97	0.39	− 0.28	0.51	0.22	1.67	2.62	4.42	0.37	4.49	0.46	0.17
3	3.67	2.79	1.78	0.19	0.23	0.77	0.21	0.47	1.61	2.35	4.09	0.42	3.76	0.61	0.26
4	3.81	2.47	1.62	1.27	0.37	0.79	− 0.82	0.40	1.51	2.08	3.26	0.46	3.33	0.90	0.42
5	4.17	2.77	1.53	0.81	− 0.26	− 0.24	0.10	0.29	1.55	3.62	3.64	0.57	2.52	1.25	0.71
6	4.19	1.98	2.04	0.15	0.26	− 0.30	0.40	0.63	1.37	3.90	3.57	1.13	0.79	1.78	2.00
7	3.85	3.77	0.84	0.89	− 0.36	0.49	0.31	0.93	1.26	2.60	4.58	1.69	0.19	2.24	3.79
8	2.78	1.70	1.59	0.31	0.01	− 0.72	− 0.28	0.43	1.05	2.06	3.67	0.64	–	–	–
9	3.42	3.12	0.92	1.49	− 0.02	− 0.70	− 0.14	1.07	1.90	2.56	4.01	1.33	–	–	–
10	4.18	1.78	1.12	− 0.26	− 0.06	0.46	0.50	1.72	1.48	1.44	4.69	1.62	–	–	–
11	4.49	2.20	1.50	0.99	0.34	0.33	0.10	0.60	1.44	2.10	4.56	0.92	–	–	–
12	3.88	2.81	0.98	0.10	0.11	− 0.12	0.73	0.91	1.65	1.79	3.41	0.65	–	–	–
13	3.97	2.31	2.01	1.26	− 0.12	0.42	0.82	0.77	2.17	2.99	3.98	0.95	–	–	–
14	4.30	2.57	1.20	0.79	0.17	− 0.18	1.01	0.73	2.71	2.34	4.15	1.83	–	–	–
15	4.63	3.79	1.54	0.24	0.54	− 0.63	0.02	0.75	2.11	3.17	3.86	2.73	–	–	–

**Figure 7:**
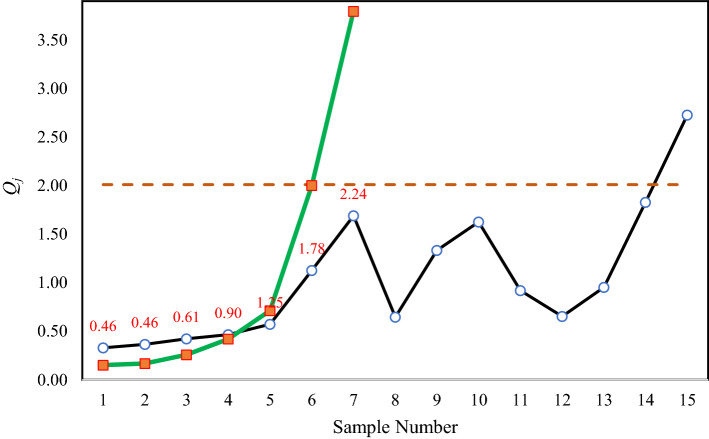
The chart statistic of ANEWMA (green) and NEWMA (black) control charts for the simulated data.

For better understanding, the computations of *Tr*_*j*_ (in the second and last sample) are illustrated here. In the second sample, *T*_*2*_ = $\frac{2.01 - 0.366}{{0.366}} = 4.49$, T*_2_ = 4.8, *Tr*_*2*_ = max(0.46,5.24–1.04 × 4.8) = 0.46 and Q*_2_ = 0.37 × 0.46 = 0.17. In a similar way, we have, *T*_*7*_ = $\frac{2.01 - 1.69}{{1.69}} = 0.19$, T*_7_ = 2.88, *Tr*_*7*_ = max(0.46,5.24–1.04 × 2.88) = 2.24 and Q*_7_ = 1.69 × 2.24 = 3.79. The charting statistic in ANEWMA and NEWMA for the above data are depicted in Fig. 7 with green and black lines, respectively. The values of *Tr*_*j*_ are shown in red.

## Conclusions

In this paper, an effective adaptive approach for improvement of the non-parametric profile monitoring in Phase II was proposed. The adaptive approach was combined with the NEWMA control chart proposed by Zou et al.^28^, to enhance the OC detection in Phase II monitoring. To compute the control limits, a simulation-based designing procedure was proposed based on the Monte Carlo theory. The results showed that the proposed method not only improved the performance of NEWMA approach in phase II, but also outperformed some of the existing control charts, such as NM, EWMA3 and other adaptive methods, for most shifts in terms of ARL criterion. In these simulations, three different scenarios (IC models) entailing polynomial, exponential and linear models have been investigated to show the robustness of the proposed scheme. In the case study, an example from the semiconductor device fabrication was implemented in which remarkable ability of the proposed method for the detection of OC shifts was demonstrated. For future research, we suggest the development of a control chart based on the combination of this approach and other adaptive control schemes. Also, obtaining the exact distribution of the chart statistics and control limits for adaptive control charts would be a novel idea.

## Data Availability

The datasets used and/or analyzed during the current study are available from the corresponding author on reasonable request.
